# Elementary school children’s recess schedule and dietary intake at lunch: a community-based participatory research partnership pilot study

**DOI:** 10.1186/1471-2458-14-156

**Published:** 2014-02-12

**Authors:** Monica Hunsberger, Paul McGinnis, Jamie Smith, Beth Ann Beamer, Jean O’Malley

**Affiliations:** 1 Public Health Epidemiology, University of Gothenburg, Box 454, Gothenburg, SE 405 30, Sweden; 2 Greater Oregon Behavioral Health, Inc, 309 E. 2nd Street, The Dalles, OR, 97058, USA; 3 Jefferson County School District 509-J, 445 SE Buff Street, Madras, OR, 97741, USA; 4 Mountain View Community Health Improvement and Research Partnership, Mountain View Hospital, Madras, OR, 97741, USA; 5 Oregon Clinical & Translational Research Institute, Oregon Health & Science University, Portland, OR 97239, USA

**Keywords:** Recess schedule, Recess before lunch, Dietary intake, Milk intake

## Abstract

**Background:**

School recess before lunch (e.g., reverse recess) has been suggested as a means to improve dietary intake and classroom behavior but limited research explores this school-based policy. This pilot study tests the impact of recess scheduling on dietary intake at school lunch.

**Methods:**

A mixed methods approach included assessment of dietary intake assessed by measured plate waste on five non-consecutive days at Madras Elementary School, Madras, Oregon, United States (n = 104 intervention; 157 controls). Subjects included primary school children in grades kindergarten, first and second. Logistic regression was used to test associations between recess timing and dietary intake. Four focus groups involving teachers and staff explored reactions to the intervention. Qualitative data was transcribed verbatim and assessed for key themes.

**Results:**

Milk consumption was 1.3 oz greater in the intervention group (5.7 oz vs. 4.4 oz); and 20% more of the intervention participants drank the entire carton of milk (42% vs. 25%, p < 0.0001). Intervention participants were 1.5 times more likely to meet the nutritional guidelines for calcium (≥267 mg, p = 0.01) and fat (≤30% of total energy, p = 0.02). Consumption of entrees, vegetables, and fruits did not differ between groups. Teachers perceived recess before lunch beneficial to classroom behavior and readiness to concentrate following lunch.

**Conclusions:**

The recess before lunch intervention yielded increased milk consumption; the nutritional and social benefits observed warrant policy change consideration. Future research should assess the impact of recess before lunch in larger districts.

## Background

Policy changes that improve school environments are recommended as a means to reduce childhood obesity [1] with nearly one-third of children and adolescents overweight or obese in the United States [2]. Broad policy recommendations, however, may not provide guidance to school districts in distinct settings (e.g., rural vs. urban). In particular, there may be large differences in resources available; monetary or otherwise.

Although the factors contributing to obesity are complex, it is understood that children are consuming too many calories and not enough nutrients [3]. Studies find that rural children are more likely to be overweight or obese than children living in metropolitan areas [4,5]. Developing approaches to tailor interventions to the unique needs of rural school settings may be critical for shaping policy to reduce child and adolescent obesity nationally as rural communities are often isolated and therefore have unique needs. Because of the unique needs in rural communities this research is based upon community-based participatory research (CBPR); research in which the community works to set the research agenda [6].

The National School Lunch Program (NSLP) sets age specific nutrient standards to ensure schools serve age appropriate, nutritious and well-balanced meals [7]. These include minimum levels for energy (664 kcal), a maximum proportion of fat (not to exceed 30%) and saturated fat (not to exceed 10%), and minimum levels of protein (10grams), calcium (286 mg), iron (3.5 mg), and vitamins A (224RE) and C (15 mg) for children in grades K-6 [7]. Therefore, it is optimal if children eat their NSLP lunch.

Recess before lunch (e.g., reverse recess) has been purported as a means for improving dietary intake and classroom behavior in children. Those in favor of recess before lunch contend that a student eager to get outside and play may rush through lunch or skip it all together, leaving them hungry later in the day [8,9]. Children increasingly skip meals and consume empty calories from snacks. As much as 27% of children’s calories come from snacks; many of which contain empty calories from added fats and sugars [8,9]. Therefore, the balanced meal provided by the NSLP may assist in displacing otherwise empty calories that may be consumed as snacks later in the day.

However, early studies on recess before lunch yielded conflicting results [10-12]. For example, there have been reports of reduced food waste when recess is offered before lunch [13,14] as well as barriers and challenges in making these changes in schools [15-17]. In January 2010, recess before lunch received mass media attention with a headline in the New York Times that read *Play, Then Eat: Shift May Bring Gains at School*[18]. However, there is limited research on recess scheduling as a means for improving dietary intake.

The impact of recess before lunch interventions may vary based on local contexts and their methods of implementation. Because of the paucity of research and conflicting findings, our CBPR group chose to investigate recess before lunch as a school-based intervention to improve nutrient intake (short term) and support the community partner’s long-term objective of reducing the number of overweight or obese children (long term). The primary objective of this study was to examine dietary intake in relation to recess scheduling in a diverse elementary school in rural Oregon using a mixed-methods approach planned by an academic-community partnership. We hypothesized children with recess before lunch would consume a greater quantity of dietary nutrients from food and drink and have fewer behavioral infractions as reported by teachers and staff than children with recess after lunch.

## Methods

The academic-community partnership responsible for this research included Oregon Health & Science University (OHSU), a diverse group of professionals from the 509-J school district in Madras, Oregon, USA including the school nurse and the food service director and the elementary school principal responsible for the intervention school, the Confederated Tribes of the Warms Springs Health and Wellness Committee, staff from both the Women, Infant and Children’s (WIC) program and Head Start, Mountain View Community Health Improvement Partnership staff, and citizen volunteers. Partnership development has been described in detail elsewhere [19]. All members of the partnership were full collaborators who identified community issues and made recommendations for interventions that would be feasible and sustainable. Before the study began, OHSU Institutional Review Board (IRB), the Portland Area Indian Health Service IRB, and the school principal approved this study. Written consent from adult participants and in the case of minors, parental written consent and the child’s assent were obtained.

### Participants

#### Quantitative plate waste

Within one elementary school (15 classes, n = 261), students were scheduled into either recess before lunch (intervention, n = 104) or recess after lunch (control group, n = 157) by classroom comprised of grade levels K-2 for the academic year that began September 14, 2009 and ended June 10, 2010. Assignment to intervention or control was distributed across grade levels and was determined by the school principal. At assignment, children ranged in percentile body mass index (BMI) from 5 to 100 with a mean BMI percentile of 70.4 ± 26.7. There was no statistical difference between recess arms (69.2 ± 26.8 vs. 71.1 ± 26.7, Pearson’s chi-square =96.1, p = 0.14).

Plate waste data was collected on five, nonconsecutive days (10-22-2009; 12-9-2009; 1-12-2010; 2-12-2010; 3-2-2010) by trained research staff using standardized measuring procedures. There were no additional measurements taken at baseline or follow-up. All students in the study ate in a common cafeteria at prescribed times, from a single-serve line. The common cafeteria is the only outlet providing food at this school. However children are permitted to bring a packed lunch from home. The school district utilizes an offer versus serve system. Students are offered a pre-portioned fruit, vegetable, starch, protein, and milk (skim, 1% fat or 1% fat chocolate) choice daily of uniform volume or weight which meets the NSLP nutrition standards. Each student is required to take a minimum of three items offered. In addition, some menu items are offered with condiments (e.g., ketchup, mayonnaise, mustard) served in individual serving packets. Students in the intervention and control groups were offered identical menu items on all five days. Research staff collected plate waste data taking into account food items offered but refused. Participants included only students participating in the NSLP; those bringing food from home were excluded. The majority of students were included as nearly 80% of students qualified for free/reduced lunch and most participated in the NSLP. We used daily attendance records to track eligible student participants for each study day and therefore participation numbers varied by day. Both intervention and control groups were allotted 20 minutes for lunch and 20 minutes for outdoor recess as a continuous 40 minute session. Lunch and recess were distributed over three periods beginning at 11:40 am, 12:00 pm, and 12:20 pm and with assignment to intervention or control.

### Instruments

#### Plate waste

Plate waste was measured using Ohaus CT1200 Portable Digital gram scales (Florham Park, NJ) and milk was measured to the nearest milliliter using a liquid measuring cup. The nutrient content of foods offered during the study period was analyzed with *NUTRIKIDS Menu Planning & Nutritional Analysis* software (Lunchbyte Systems, Inc, Rochester, NY, 2001). At the start of measurement days, we weighed three servings of each standard, pre-portioned menu item to establish an average pre-consumption gram weight. Milk was served in a standard single-serving eight ounce (240 milliliters) container. Plate waste data were collected using standard procedures previously described [20-23]. In brief, on the measurement days trash bins were removed and children placed their discarded lunch plates onto trays labeled by classroom. Lunch plates on the intervention days were discretely color and number coded on the bottom to represent classroom and student identification number. Consumption was calculated by subtracting the remaining fruit, vegetable, entrée, and condiments from the pre-consumption weight. Researchers worked in pairs, verifying each weight and liquid measure by subject. USDA sets standards for the school lunch meal and therefore we assessed nutrient consumption against established criteria for the lunch meal.

All quantitative analyses were completed using SAS 9.2. The nutrient content of the consumed portion was calculated by multiplying the percent of the portion consumed by the nutrient content of the standard portion derived from USDA nutrient database calculated with *NUTRIKIDS* software. The associations between recess timing and the probability of drinking an entire serving of milk and the probability of meeting USDA guidelines for calories, protein, iron, calcium. The association of meeting dietary recommendations for vitamins A and C, fat, and saturated fat were tested using logistic regression models with generalized estimating equations to take into account the within student correlations and include the testing day as a fixed effect as any variation that is attributed to the day of measurement represents quantities that were non-random. The consumption of sodium was too variable over the five menus to allow model convergence so the estimation of meeting the recommendations for sodium intake utilized separate models for each day.

#### Qualitative focus groups

Following the completion of plate waste data collection, we held focus groups with teachers (2 sessions with 7 teachers and 8 teachers respectively for a total of 15 teachers), food service personnel (1 session with 5 employees), and educational aides (EA) (1 session with 6 educational aides) in May 2010. Educational aides assist on the playground and in the cafeteria and hence their participation was relevant to the research question. Focus groups were conducted with a semi-structured script to assess reactions to recess first in a manner that allowed for group dynamics among work groups but not across work groups due to the unique job responsibilities. Focus groups were digitally recorded and transcribed verbatim for analyses. Qualitative data were analyzed by method of constant comparison which stems from Grounded Theory [24] but has also been used to analyze focus group content [25]. Three researchers independently coded for main themes. Following the individual theme coding consensus was reached regarding key themes.

#### RATS guidelines

The authors confirm that this study adheres to the RATS guidelines on qualitative research reporting.

## Results

### Plate waste

**Figure 1:**
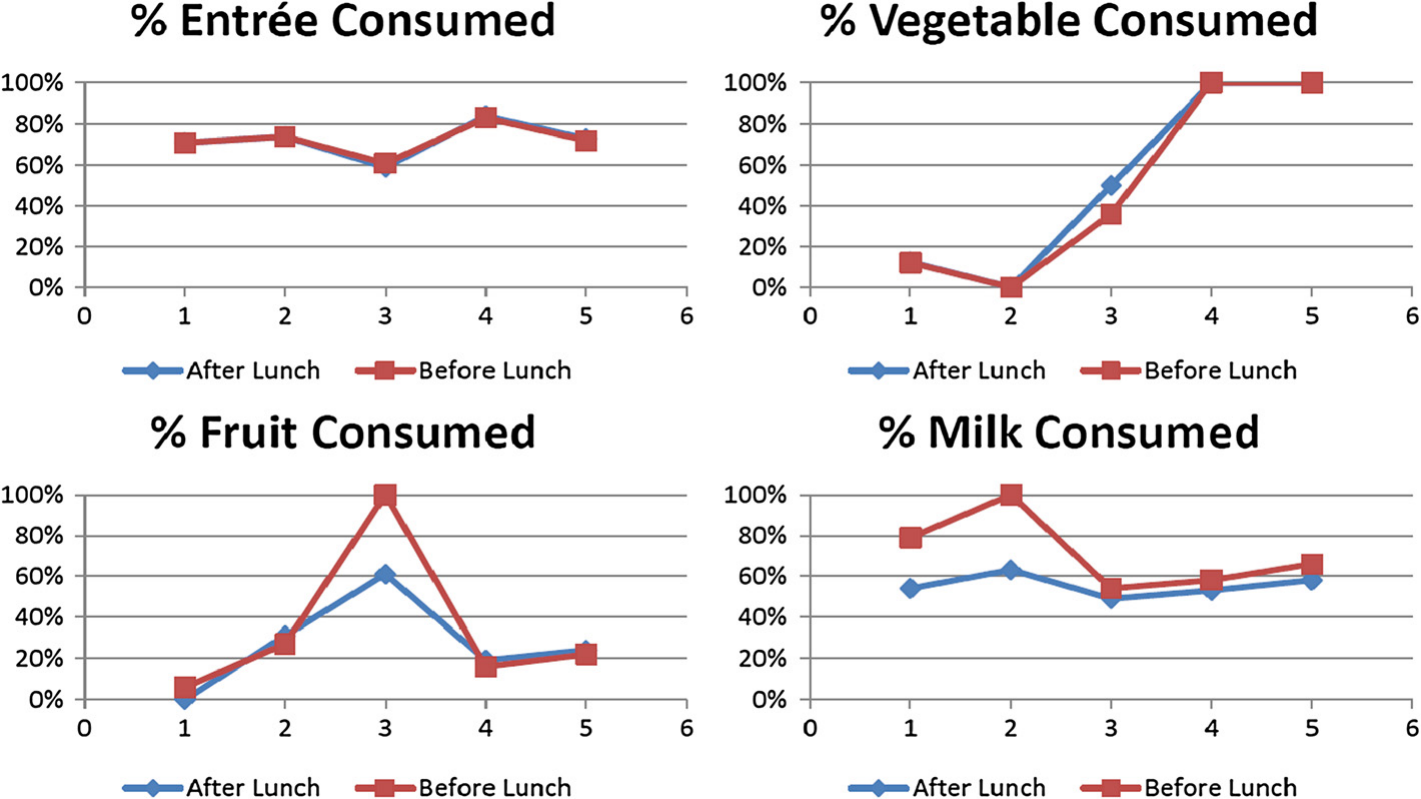
Student food consumption by day and recess order (median).

The median percent of the standard portions of entrées, vegetables, and fruits consumed varied from day to day but did not differ by recess group (Figure 1). On all 5 study days, the median percent of milk consumed was higher for students in the intervention group (e.g., recess before lunch) as compared to the control group (see Figure 1). Students in the intervention group were almost 20% more likely to drink the entire carton of milk as compared to controls (42% vs. 25%, p < 0.0001).

**Table 1:** Association of recess order with probability of consumption meeting nutritional guidelines

**Nutrient**	**Probability modeled**	**Mean probability before lunch**	**After lunch**	**OR (95% CI)**	**p-value (two sided)**
Calories^1^	Intake > =475 cal				
	(75% guideline)	27%	23%	1.27 (0.9 – 1.8)	0.169
Protein	Intake > =9 g	86%	84%	1.22 (0.9 – 1.7)	0.270
Iron	Intake > =3.3 mg	15%	13%	1.20 (0.8 – 1.7)	0.304
Calcium	Intake > =267 mg	68%	59%	1.49 (1.1 – 2.0)	0.015*
Vitamin C^2^,^3^	Intake > =10 mg				
	(66% guideline)	42%	38%	1.23 (0.9 – 1.7)	0.231
Vitamin A RE	Intake > =150				
	(75% guideline)	40%	36%	1.19 (0.9 – 1.6)	0.268
Fat	Fat calories < =30% total calories	15%	11%	1.5 (1.1 – 2.1)	0.021*
Saturated fat	Saturated fat calories < =10% total calories	0%	0%		
Sodium^4^ Day 1	Sodium < =1200 mg	83%	93%	0.38 (0.2 – 0.9)	0.024*
Sodium^4^ Day 2	Sodium < =1200 mg	49%	53%	0.86 (0.5 – 1.4)	0.556
Sodium^4^ Day 3	Sodium < =1200 mg	61%	55%	1.26 (0.8 – 2.1)	0.348
Sodium^4^ Day 4	Sodium < =1200 mg	49%	53%	0.9 (0.5 – 1.4)	0.556
Sodium^4^ Day 5	Sodium < =1200 mg	100%	99%		

^1^Too few students met 100% of guideline to model this probability, so a lower cut-off was used.

^2^Excludes Day 3 because no students met guideline for Vitamin C on Day 3 (maximum intake = 2.96 mg).

^3^Too few students met guideline of 10% to model this probability, so a higher cut-off was used.

^4^Too variable by day to combine days into a single model.

*p < 0.05.

OR = odds ratio.

Recess order was not significantly associated with the probability of a student meeting the USDA nutritional standards for total calories, protein, Vitamin C, Vitamin A or iron. However, students in the intervention were 1.5 times more likely to meet the nutritional guidelines for calcium intake (≥ 267 mg, p = 0.01) and total fat intake (≤ 30% of total calories, p = 0.02). All students exceeded saturated fat guidelines for the days examined (Table 1). Vegetable intake on measurement days one and two was low when sweet potatoes and salad with ranch dressing were served. On measurement day three, when carrots with ranch dressing were served, intake increased for both groups. On measurement days four and five vegetable intake was highest when potato rounds and tater tots were served. Similarly, fruit intake was poor when students were served unsweetened Marionberries from frozen berries, quartered oranges with peel remaining, kiwi halves with skin on and canned peaches (days one, two, four, and five respectively). Some students were unfamiliar with kiwis and did not know how to eat them, biting into fuzzy kiwi skins versus peeling them. Fruit intake was highest when pineapple with cottage cheese was served.

### Qualitative findings

We hypothesized children with recess before lunch would have fewer behavioral infractions as reported by teachers and staff than children with recess after lunch. Our findings from four focus groups with teachers, kitchen staff and educational aides support this.

#### Calmer

Classroom teachers with students in the intervention group consistently noted that students came back to the classroom more calm and ready to begin lessons. They stated the students were better able to settle into their work when transitioning from the lunchroom rather than the playground as illustrated by the following quotes:

*“They seem much more settled when they've had the recess, and then the lunch.”* [Focus Group 1; Teacher].

*“They seem to settle into academics much faster”.* [Focus Group 2”; Teacher]

#### Scheduling considerations

The staff and teachers also identified unanticipated consequences of recess before lunch. For example, one group of students had recess, lunch, and then physical education class before coming back to their classroom. This teacher described her students as “*tuckered out.”*

In response, students were offered teacher and parent supported snacks, which are given at the discretion of the teacher at inconsistent times. A teacher in focus group one stated: “*I used to give a snack in the morning but now I give it in the afternoon.”*

This academic year there were more classes and some of the students ate lunch as late as 12:25 pm. Two teachers in focus group session two both cited having five hours or more between meals but they handled this differently. One teacher said five hours is “*just too long to go*” noting a classroom snack is appropriate while the other teacher said *“I just make them wait hungry because they eat their lunch better.”* Teachers consistently stated the kids are too hungry when lunch is at noon or later. Further, students arrive at various times in the morning with differing home schedules. Some students arrive as early as 7:30 am and if they eat breakfast at home as opposed to at school it will likely be greater than five hours between their morning meal and lunch meal. The interval between meals was cited as a problem irrespective of lunch order however, one teacher said, “*some of the kids, when they are going out* (to recess)*, are like, when do we get lunch?”*

In focus group session four, foodservice staff perceived no difference in staffing or preparing menu items during the intervention and identified the issue of timing and meal spacing as a problem regardless of recess order. As stated by one of the food service staff: “*Those little guys, from 8:00 am when they eat breakfast until 12:30 pm when they're just sitting down at lunch is a long time for a kindergartener.”* It was suggested that a larger cafeteria would help ease this problem.

#### Unanticipated benefits and needs

As a consequence of the intervention, children were outside in smaller numbers and in the lunch room in smaller numbers; in focus group three the educational aides found this to be much more manageable. One stated, “*I have to say I've had a couple kids tell me, during lunch time, this is so much more relaxing than before.”* Staffing increased to manage the intervention but this increase would not be permanent if recess before lunch became the standard. “*If everybody was doing the same thing they could probably get by with three assistants instead of four.”* While discussing pros and cons of scheduling recess before lunch an educational aide stated, *“I think some of our kids in 1st grade, who are eating lunch first and who really like to go out to recess, they see the first ones lining up, then they're like, &oh a bite or two and I'm ready to go.’ They still have time but they are ready to go to recess.”* The educational aides expressed concern over “*slow-eaters*” who in the past have been allowed to keep eating and delay recess. When recess is first they must return to class on time.

## Discussion

Students in the intervention group drank significantly more low-fat milk than controls and were therefore more likely to meet recommendations for calcium and fat intake. While the milk consumption was greater on each of the five observation days, days one and two represent the majority of the difference. It is noteworthy that on day 1 and 2 when milk consumption was high the fruit and vegetable consumption was low. The increased milk consumption may be reflecting hunger or the need for additional calories when other items served were less palatable. There were no significant differences in food intake. Fruit and vegetable intake varied by acceptability of what was offered for both groups with little impact due to recess timing. Since these are isolated days, our findings suggest that children in this age group eat the foods they are familiar with, like, or prefer.

Our increased milk consumption finding is unique but warrants further investigation since days three, four and five represent a small difference in consumption between the intervention group and controls. However, this finding is important given the United States Department of Agriculture’s (USDA) attention to low calcium intake in the Dietary Guidelines for Americans 2010 [3]. Further, this finding is supported by a recent publication which found milk and milk products contribute significantly to micronutrient density in U.S. diets [26]. While others have found changes in food consumption, we did not find a significant difference [13,14,27]. We cannot explain the non-significant difference in our dietary intake findings but we could certainly see that all children had preferred menu items that were more readily consumed by both intervention and control groups. This finding has implications for those planning school meals as it may be of value to offer foods that meet nutritional guidelines and are accepted by children in the target age group.

The barriers encountered were similar to those stated in previous studies [15,16]. The first barrier was scheduling. Even with a willing and supportive school principal, rearranging the schedule was a challenge. The kitchen staff and educational aides worked slightly longer hours to accommodate the schedule changes and this increased staffing costs. Hand-washing for the recess before lunch group was also an issue; as others have identified [15-17]. The cafeteria does not have hand-washing sinks and therefore sanitizing hand wipes served in place of hand-washing. The wipes cost $300.00 for the intervention period and some parents did not view hand wipes as an adequate replacement to hand-washing.

This study is not without limitations. First, this intervention was held in one rural elementary school and therefore cannot be generalized to larger, metropolitan schools. Second, children eat pre-portioned foods from a single service line at this school while many schools allow children to self-serve from a salad bar, hot food buffet or milk dispensers. We were not able to examine how self-service might influence our findings. Additionally, because the school is a low-income school, most children participate in the NSLP at no cost to their families. Finally, there was variation in the children present at each measurement day due to absences that may introduce bias.

Despite these barriers and limitations, we have contributed to a under explored school-based policy that has practical significance. While our study was conducted in just one elementary school we did have power to detect differences. Future research should examine recess before lunch as an intervention in larger districts across multiple schools. Further, based on our unanticipated lessons, namely timing and teacher/parent supported snacks; future research should consider these factors. Our milk consumption finding is important in light of the fact that for many children it is an important source of calcium. Our qualitative research findings do support the stated hypothesis as children returned to the classroom calmer and ready to begin lessons. Based upon our findings school districts should consider offering recess before lunch in elementary schools.

## Conclusions

Our findings from one rural elementary school warrant policy change consideration. The recess before lunch intervention yielded increased milk consumption and hence increased calcium intake. The nutritional and social benefits observed indicate that changes to the school environment can positively impact nutritional intake. Future research should assess the impact of recess before lunch in larger districts.

## Competing interests

The authors declare that they have no competing interests.

## Authors’ contributions

MH conceptualized the study and collected data with co-authors and community partners. PM, BAB and JS assisted in conceptualizing the study and collecting data, JO completed all statistical analyses. All authors worked on manuscript preparation and final edits. All authors read and approved the final manuscript.

## Pre-publication history

The pre-publication history for this paper can be accessed here:

http://www.biomedcentral.com/1471-2458/14/156/prepub
